# Serum adropin levels as a potential biomarker for predicting diabetic kidney disease progression

**DOI:** 10.3389/fendo.2025.1511730

**Published:** 2025-02-07

**Authors:** I-Wen Chen, Cheng-Wei Lin, Chia-Ni Lin, Szu-Tah Chen

**Affiliations:** ^1^ Division of Endocrinology and Metabolism, Department of Internal Medicine, Chang Gung Memorial Hospital, Linkou Medical Center, Taoyuan, Taiwan; ^2^ College of Medicine, Chang Gung University, Taoyuan, Taiwan; ^3^ School of Medicine, National Tsing Hua University, Hsinchu, Taiwan; ^4^ Department of Laboratory Medicine, Chang Gung Memorial Hospital, Linkou Medical Centre, Taoyuan, Taiwan; ^5^ Department of Medical Biotechnology and Laboratory Science, College of Medicine, Chang Gung University, Taoyuan, Taiwan; ^6^ Division of Endocrinology and Metabolism, Department of Internal Medicine, New Taipei Municipal TuCheng Hospital, New Taipei City, Taiwan

**Keywords:** adropin, biomarkers, diabetic kidney disease, diabetes, kidney disease

## Abstract

**Background:**

To investigate the value of serum adropin in predicting chronic kidney disease (CKD) progression in subjects with type 2 diabetes (T2D).

**Materials and methods:**

Serum adropin levels were measured in normal control and T2D patients with various stage of CKD. CKD progression was defined as ≥ 30% decline from the baseline estimated glomerular filtration rate. Logistic regression analysis was applied to assess the association between adropin levels and CKD progression.

**Results:**

The study included 58 subjects with T2D (18 early CKD and 40 advanced CKD) and 9 subjects without diabetes (control). Subjects with T2D had significantly higher adropin levels than controls (6393.10 ± 1611.84 vs. 3470.30 ± 1284.41 pg/ml; *P* < 0.001). Meanwhile, T2D patients with advanced CKD had higher adropin levels than those with early CKD (6848.89 ± 1287.04 vs. 5380.25 ± 1826.44 pg/ml; *P* = 0.003). Among T2D patients, subjects experienced CKD progression had higher adropin levels than those without (7520.15 ± 843.21 vs. 6151.16 ± 1661.61 pg/mL, *P* =0.003). Thus, adropin predicts CKD progression in T2D patients with 86% sensitivity and 70% specificity at 6872.24 pg/ml cutoff value. The association with CKD progression was still significant after adjusting for age, gender and body mass index (adjusted odds ratio = 27.188, 95% confidence interval 1.415-522.527, *P* =0.029).

**Conclusions:**

The above findings suggest that serum adropin could be applied as a potential biomarker for predicting CKD progression in subjects with T2D. Further research is needed to validate these results and explore the underlying mechanisms.

## 1 Introduction

Diabetic nephropathy is a worldwide-leading cause of end-stage kidney disease resulting in dialysis or renal transplantation in about 20–40% of patients with diabetes patients (1). Additionally, such patients with diabetic kidney disease (DKD) might also have exceptionally high rates of cardiovascular morbidity and mortality (2); furthermore, along with glycemic control, the renin-angiotensin-aldosterone system (RAAS) has also been shown as associated with the progression of DKD, promising advancements in chronic kidney disease (CKD) management in type 2 diabetes mellitus (T2D) including angiotensin-converting enzyme inhibitors (ACEi), angiotensin II receptor blockers (ARB), sodium-glucose cotransporter-2 inhibitors (SGLT2i), and nonsteroidal mineralocorticoid receptor antagonists (MRA).

Microalbuminuria is considered an important predictor of progression to proteinuria or nephropathy; however, its usefulness for early detection and monitoring of CKD progression is limited (3, 4). In certain cases, such as in patients with T2D, CKD can be observed even in the absence of albumin excretion, indicating that albuminuria alone is inadequate for monitoring diabetic nephropathy (3). This inconsistency has been documented in studies showing normal renal function in patients with microalbuminuria, while those with normoalbuminuria have renal disorders (5, 6). These findings highlight the limitations of using albuminuria or serum creatinine alone as early indicators of renal dysfunction; therefore, novel biomarker potential for early detection of patients in risk for CKD progression is warranted.

Adropin is a newly identified peptide hormone expressed in the liver and brain, and has been implicated in processes such as energy homeostasis and lipid metabolism (7). Demographic factors such as gender, age and body mass index (BMI) might affect the serum levels of adropin (8). Observation studies have reported decreased adropin levels in patients with obesity, metabolic syndrome, and type 2 diabetes, suggesting the correlation of adropin and insulin resistance (9); indeed, treatment with adropin lowered blood glucose levels and improved insulin resistance in type 2 diabetic animal models (10). Interestingly, conflicting serum adropin levels have been reported in some patients with T2D (11, 12) lead to the role of adropin plays in diabetes becoming much more complex as previously assumed.

In addition to its expression in the liver and brain, adropin has also been detected in the kidney, including glomeruli, peritubular interstitial cells, and peritubular capillary endothelial cells (13). Interestingly, adropin immunoreaction was enhanced in the kidney of streptozotocin (STZ)-induced diabetic rats compared with the controls (13); and subsequently, adropin was found to reduce the expression levels of inflammatory markers like tumor necrosis factor-alpha (TNF-*α*) and interleukin 6 (IL-6) in diabetic rats (10). On considering that inflammation plays important roles in the development of diabetic nephropathy, these findings suggest that adropin could exert a protective effect against the development of diabetic nephropathy through its anti-inflammatory actions; accordingly, given the evidence presented, this study aimed to examine serum adropin levels in subjects with diabetes at various stage of CKD and explore the potential application of adropin levels as an adjuvant indicator of CKD progression in individuals with T2D.

## 2 Materials and methods

### 2.1 Subjects and data source

We enrolled subjects with T2D being regularly followed-up at the Chang-Gung Memorial Hospital (CGMH), a tertiary hospital in Northern Taiwan, since September 2021. The diagnosis of T2D fulfilled the American Diabetic Association (ADA) criteria and all patients received antidiabetic drugs to control hyperglycemia. Subjects with type 1 diabetes, severe cardiovascular diseases, malignant diseases, acute infection, hepatic or endocrine diseases were excluded. The non-diabetic controls enrolled from the same clinic were supervised by the endocrinologists and assured to have normal blood glucose and no history of diabetes. This study was conducted in accordance with the Declaration of Helsinki and was approved by the Ethics Committee on Research of the Institutional Review Board (IRB approval number: 201900988B0) of CGMH. Informed consents were obtained from all participants with their full appreciations of the study.

### 2.2 Baseline measurement and covariates

Clinical information including age, gender and the date of diagnosis as well as laboratory data (serum hemoglobin, hematocrit, creatinine, glycated hemoglobin (HbA1c), sodium, potassium, osmolality, urine osmolality, urine creatinine, urine sodium, and urine albumin creatinine ratio) were collected. Participants were followed-up until May 31st, 2023, and the primary outcome (endpoint) was CKD progression, being a decline of eGFR at least 30% from the baseline accordingly (14).

### 2.3 Calculation of estimated glomerular filtration rate and urinary albumin creatinine ratio

The estimated glomerular filtration rate (eGFR) was calculated using the Modification of Diet in Renal Disease (MDRD): i.e. 175 x serum creatinine (exp[-1.154]) x age (exp[0.203] x (0.742 if female) (15). We manually measured spot urinary albumin creatinine ratio (UACR) by dividing spot urinary albumin to urinary creatinine, with results were expressed in mg/g to meet the standard guidelines. According to the Kidney Disease Improving Global Outcomes (KDIGO) guidelines, individuals at risk category of CKD were classified into five eGFR and three albuminuria categories (1). The eGFR categories were defined as follows: grade 1: eGFR > 90 mL/min/1.73 m^2^; grade 2: 60 mL/min/1.73 m^2^< eGFR <90 mL/min/1.73 m^2^; grade 3: 30 mL/min/1.73 m^2^≤ eGFR <60 mL/min/1.73 m^2^; grade 4: 15 mL/min/1.73 m^2^ ≤ eGFR<30 mL/min/1.73 m^2^ and grade 5: eGFR < 15 mL/min/1.73 m^2^. Advanced chronic kidney disease (CKD) was defined as having an estimated glomerular filtration rate (eGFR) of less than 60 ml/min per 1.73 m², indicating significant reduction in kidney function, while in contrast, early CKD was characterized by a preserved eGFR of greater than 60 ml/min per 1.73 m², suggesting relatively normal kidney function. End-stage renal disease (ESRD) was defined as initiation of chronic hemodialysis or peritoneal dialysis.

### 2.4 Analysis of serum adropin levels

Serum adropin levels were measured in duplicate using an enzyme-linked immunosorbent assay (ELISA) kit from Elabscience (Houston, TX, USA). The standard curve for adropin ranged from 12.5 pg/mL to 800 pg/mL, with a sensitivity of 7.5 pg/mL, while the intra-assay coefficient of variation (CV) for adropin was less than 10% in terms of functional sensitivity and the inter-assay CV was less than 15%.

In short, serum was obtained from a total of 10 mL of venous blood taken in the morning after at least 12 h fasting and stored at − 80°C. Aliquots of 10 μl of 5-fold diluted serum sample was added into wells coated with adropin antibodies, followed by the addition of 100 μl of HRP-conjugated reagent. After incubation for 60 min at 37°C, unbound Streptavidin-HRP was washed away, then the reaction was terminated by addition of acidic stop solution and the absorbance was measured at 450 nm. The five-parameter logistic fitted standard curve for calculating the concentration of adropin was generated from the Arigo Biolaboratories website (https://www.arigobio.cn/ELISA-calculator).

### 2.5 Statistical analyses

Data were expressed as means ± standard deviation or median (interquartile range). The differences of characteristics between three groups of T2D patients and control subjects were compared using Chi-square tests, or Mann-Whitney U test with Logistic regression analysis being used to determine the risk factors for developing T2D and DKD. The correlations between serum adropin and other parameters were analyzed by Pearson correlation analysis, and multiple linear regression analysis was used to determine the contribution of various factors to serum adropin. *P* values less than 0.05 were considered statistically significant. Potential predictors of CKD progression in the bivariate analyses (*P* < 0.05) were tabulated and were analyzed by logistic regression (probability of entry *P* < 0.05). Binary logistic regression was employed for multivariate analysis of risk factors causing decline in renal function. In addition, receiver operating characteristic curve (ROC) were delineated, followed by stepwise regression analysis of the role of adropin in the differential diagnosis of CKD progression. All data analyses were conducted using SPSS version 26 (IBM SPSS Inc., Chicago, IL).

## 3 Results

### 3.1 Clinical characteristics of T2D patients and controls

A total of 58 eligible T2D patients were enrolled in this study: 40 were advanced CKD, with twenty-three in stage 3, seven in stage 4, and ten in stage 5 (including three under regular hemodialysis). The remaining 18 subjects with diabetes had eGFR ≥ 60 mL/min/1.73 m^2^ and were classified as early CKD. None of these patients had pre-existing episodes of major adverse cardiovascular events. We also included a control group of 9 subjects without diabetes. The demographic, clinical and biochemical data of subjects with diabetes both with advanced and early CKD, as well as the control group are presented in **Table 1**.

**Table 1:** Demographic and clinical characteristics of T2D patients with early and advanced kidney disease and controls.

	DM (n=58)		*P* value (DM with advanced CKD vs. DM with early CKD)	Control (n=9)	*P* value (DM vs. control)
	DM with advanced CKD (n=40)	DM with early CKD (n=18)			
Age (years)	67.10 ± 10.13	52.89 ± 12.95	<0.001*	56.44 ± 11.18	0.084
Gender (Male), n (%)	27 (67.5%)	11 (61.1%)	0.636	4 (44.4%)	0.224
BMI (kg/m2)	25.34 ± 3.66	27.57 ± 6.01	0.254	25.32 ± 3.11	0.881
DM duration (years)	13.50 ± 7.21	9.39 ± 7.46	0.025*		
Osmolality (mosm/KgH2O)	304.29 ± 6.00	296.50 ± 2.68	<0.001*	294.67 ± 5.15	0.003*
HbA1c (%)	7.38 ± 1.34	8.03 ± 2.14	0.449	5.58 ± 0.47	<0.001*
BUN (mg/mL)	37.65 ± 17.26	15.94 ± 2.96	<0.001*	14.90 ± 4.22	0.001*
Cr (mg/dL)	2.83 ± 2.26	1.21 ± 1.81	<0.001*	0.70 ± 0.20	<0.001*
eGFR (mL/min/1.73m^2^ )	30.57 ± 15.40	101.46 ± 37.55	<0.001*	104.77 ± 28.91	<0.001*
eGFR change (ml/min/1.73 m^2^ per year)	-4.58 ± 6.87	-8.74 ± 18.86	0.614		
Percentage of eGFR change (%)	-13.22 ± 26.83	-6.52 ± 15.71	0.175		
Hemoglobin (g/dL)	11.86 ± 2.00	14.54 ± 1.75	<0.001*	13.72 ± 1.80	0.266
Hematocrit (%)	36.59 ± 5.82	43.58 ± 5.08	<0.001*	41.29 ± 5.36	0.331
Na (mEq/L)	140.40 ± 2.73	140.22 ± 2.46	0.728	142.00 ± 1.80	0.053
K (mEq/L)	4.63 ± 0.63	4.48 ± 0.40	0.328	4.11 ± 0.36	0.010*
Adropin (pg/ml)	6848.89 ± 1287.04	5380.25 ± 1826.44	0.003*	3470.30 ± 1284.41	<0.001*
Urine ACR (mg/gm)	1351.22 ± 1912.63	368.93 ± 758.81	0.001*	7.56 ± 4.27	<0.001*
Use of OAD					
Metformin, n (%)	17 (42.5%)	17 (94.4%)	<0.001*		
Sulfonylurea, n (%)	27 (67.5%)	9 (50.0%)	0.204		
TZD, n (%)	9 (22.5%)	5 (27.8%)	0.450		
DPP4 inhibitor, n (%)	20 (50.0%)	5 (27.8%)	0.114		
GLP-1R agonist, n (%)	8 (20.0%)	4 (22.2%)	0.551		
SGLT2 inhibitor, n (%)	7 (17.5%)	8 (44.4%)	0.035*		
Insulin injection, n (%)	21 (52.5%)	6 (33.3%)	0.176		
Use of anti-hypertensive agent, n (%)	36 (90%)	10 (55.6%)	0.005*	3 (33.3%)	0.009*
Use of statin, n (%)	27 (67.5%)	9 (50.0%)	0.204	2 (22.2%)	0.034*

Continuous variables presented as mean ± standard deviation or n (%).

Mann-Whitney U test was used for continuous variables and Chi-Squared test or Fisher exact test was used for categorical variables.

*Denotes P value < 0.05.

DM, diabetes mellitus; CKD, chronic kidney disease; BMI, body mass index; HbA1c, glycated hemoglobin A1c; BUN, blood urea nitrogen; Cr, creatinine; eGFR, estimated glomerular filtration rate; ACR, albumin to creatinine ratio; OAD, oral antidiabetic drug; TZD, Thiazolidinedione; DPP4, dipeptidyl-peptidase-4; GLP-1R, glucagon-like peptide-1 receptor; SGLT-2, sodium glucose co-transporters 2.

The mean age of subjects with diabetes was 62.69 (± 12.82) years old and mean duration of diabetes was 12.22 (± 7.47) years. The average HbA1c was 7.58 (± 1.64) %; the average eGFR was 52.57 (± 40.95) mL/min/1.73 m^2^ and UACR was 1029.74 (± 1684.16) mg/g, and the average concentration of adropin was 6292.10 (± 1611.84) pg/ml. In summary, the subjects with diabetes had higher levels of blood osmolality, HbA1c, blood urea nitrogen (BUN), creatinine, potassium, adropin and UACR, as well as lower levels of eGFR as compared to the control group (**Table 1**).

In the subjects with diabetes, patients with advanced CKD were older (67.10 ± 10.13 vs. 52.89 ± 12.95 years, *P* < 0.001), sustaining a longer duration of diabetes (13.50 ± 7.21 vs. 9.39 ± 7.46 years, *P* = 0.25), and had significantly lower eGFR (30.57 ± 1./40 vs. 101.46 ± 37.55 mL/min/1.73m^2^, *P* < 0.001). Biochemically, patients with advanced CKD had higher blood osmolality (304.29 ± 6.00 vs. 296.50 ± 2.68 mosm/KgH2O, *P* < 0.001), elevated BUN (37.65 ± 17.26 v.s.15.94 ± 2.96 mg/mL, *P* < 0.001), increased creatinine (2.83 ± 2.26 vs. 1.21 ± 1.81 mg/mL, *P* < 0.001), higher adropin concentration (6848.89 ± 1287.04 vs. 5380.25 ± 1826.44 pg/mL, *P* = 0.003), elevated UACR (1351.22 ± 1921.63 vs. 368.93 ± 758.81 mg/g, *P* = 0.001), and lower hemoglobin (11.86 ± 2.00 vs. 14.54 ± 1.75 g/dL, *P* < 0.001).

### 3.2 Serum adropin concentrations

**Figure 1:**
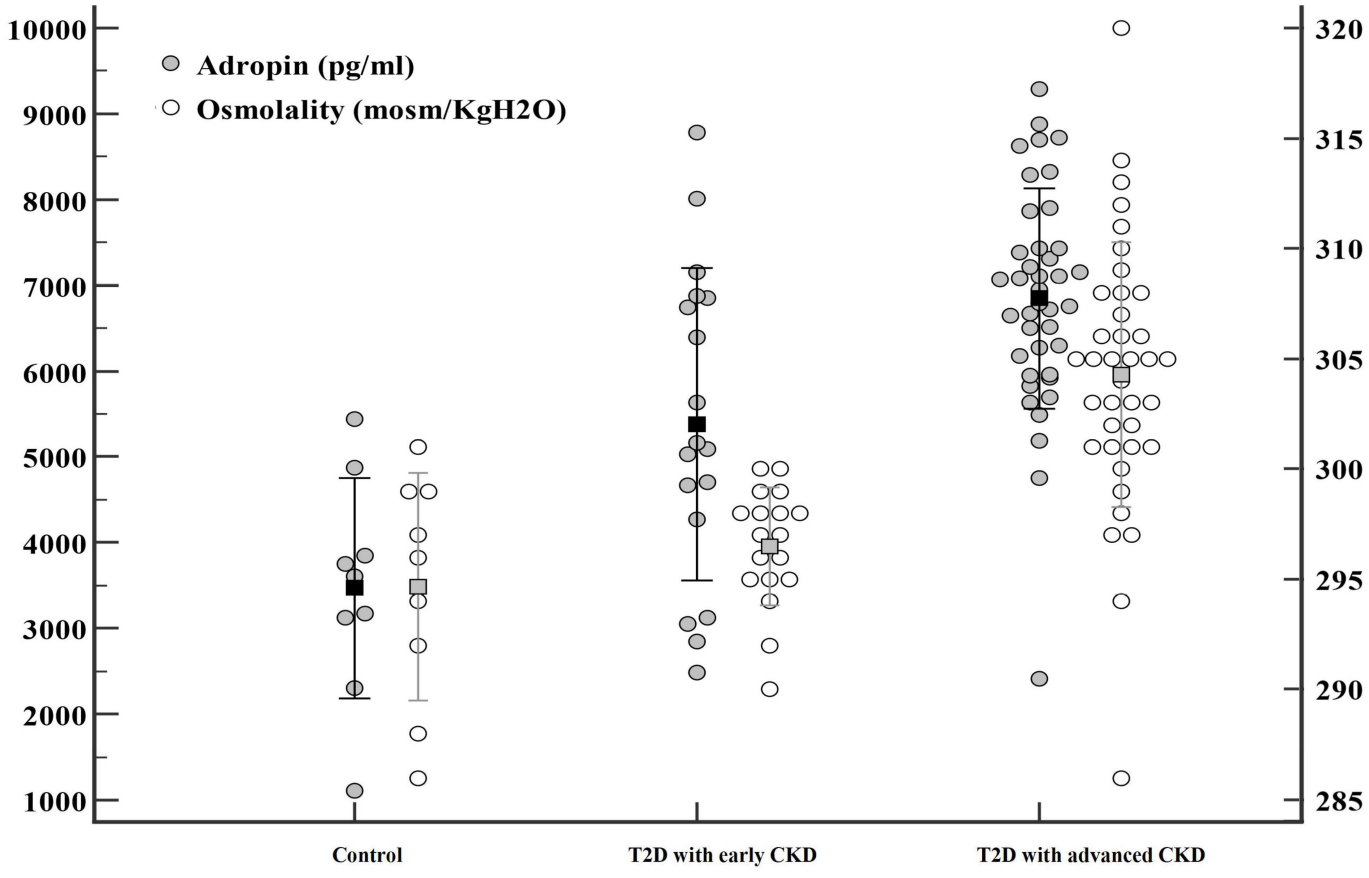
Significant differences were observed in adropin (●) and osmolality (○) levels among three groups: patients with type 2 diabetes mellitus (T2D) with advanced chronic kidney disease (CKD), patients with T2D with early CKD, and controls.

Serum adropin levels as presented in **Table 1** and **Figure 1**, were significantly higher in subjects with diabetes compared to the control group (T2D: 6393.10 ± 1611.84 pg/mL; control: 3470.30 ± 1284.41 pg/mL; *P* < 0.001). Additionally, significantly higher adropin levels were also found in T2D subjects with advanced CKD compared to those with early CKD (6848.89 ± 1287.04 pg/mL vs 5380.25 ± 1826.44 pg/mL; *P* =0.003). After adjusting for osmolality, GFR, UACR, use of anti-hypertensive agents and use of statins, the serum adropin level showed borderline significance (adjusted odds ratio (aOR) 1.001, 95% confidence interval (CI) 1.000-1.003, *P* = 0.096) between subjects with diabetes and controls.

### 3.3 Correlation of adropin levels with clinical parameters

**Figure 2:**
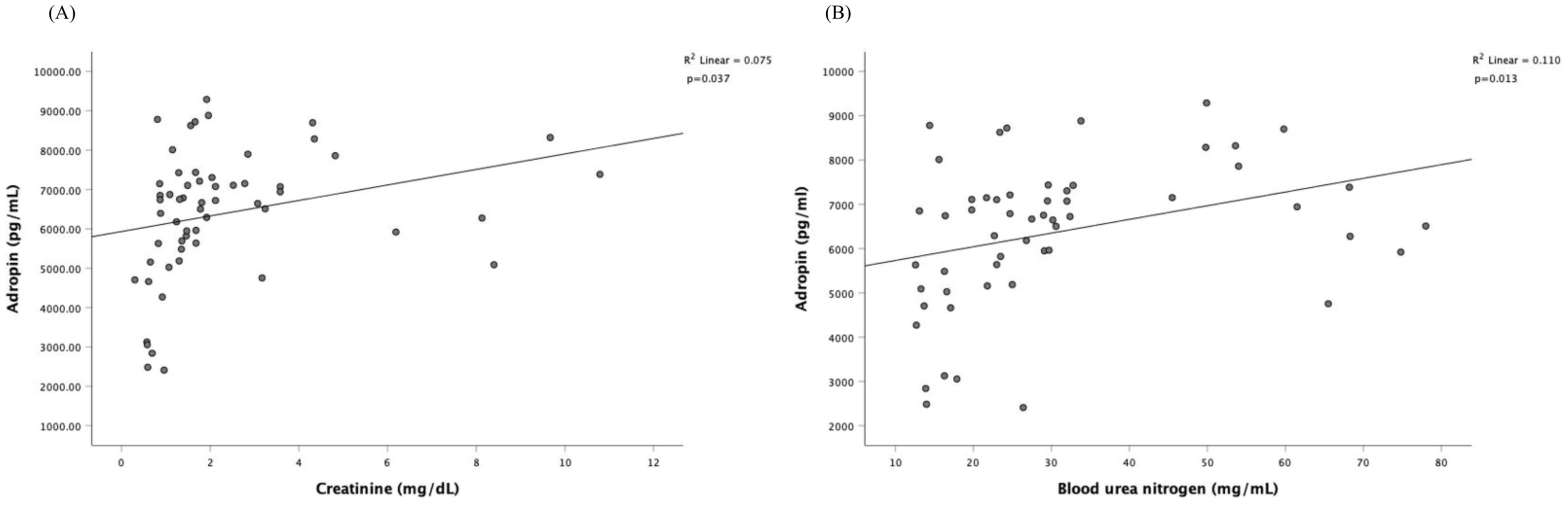
Pearson correlation analysis among serum adropin levels and renal parameters. The correlation is depicted between serum adropin levels and creatinine in panel **(A)**, between adropin levels and blood urea nitrogen in panel **(B)**.

Since serum adropin was associated with diabetic control and complications, we performed univariate and multivariate linear regression analyses in subjects with diabetes (**Table 2**) to evaluate the plausible associations of adropin levels with other variables. The unadjusted univariate linear regression analysis showed significant association between adropin levels and male gender (*P* < 0.001), osmolality (*P* =0.047), BUN (*P*=0.013), creatinine (P = 0.038), eGFR (*P* < 0.001) and use of anti-hypertensive (+/-diuretic) agents (*P* =0.007); however, age, BMI, diabetic duration, HbA1c, UACR, use of SGLT2 inhibitors and statins showed no significant association. Multivariate linear regression demonstrated that only male gender (*P <*0.001) and eGFR (*P <*0.001) remained strongly associated with adropin level, whereas use of SGLT2 inhibitors and use of anti-hypertensive agents exhibited borderline significance. **Figure 2** illustrates the Pearson correlation analysis between serum adropin levels and renal parameters.

**Table 2:** Univariate and multivariate linear regression analyses of the association of adropin levels with age, gender, BMI, osmolality, BUN, Creatinine, eGFR, Urine ACR, use of SGLT2 inhibitor, anti-hypertensive agent, and statin.

	Univariate Linear Regression			Multivariate Linear Regression		
	B Coefficient	SD	*P* value	B Coefficient	SD	*P* value
Age (per year)	21.740	-11.421-54.900	0.194			
Gender (Male)	1793.471	1032.310-2554.631	<0.001*	1507.846	829.788-2185.903	<0.001*
BMI	-49.042	-143.646-45.562	0.303			
Diabetic duration	13.473	-44.144-71.091	0.641			
Osmolality	68.744	1.070-136.419	0.047*	23.382	-36.371-83.135	0.435
HbA1c	81.931	-180.090-343.951	0.534			
BUN	30.916	6.672-55.160	0.013*			
Creatinine (mg/dL)	196.981	12.139-381.824	0.037*			
eGFR (mL/min/1.73m^2^)	-19.523	-28.671--10.374	<0.001*	-16.366	-25.769- -6.964	0.001*
Hemoglobin	-80.601	-271.188-109.986	0.400			
Hematocrit	-32.874	-100.315-34.567	0.333			
Urine ACR	0.221	-0.038-0.480	0.093			
Use of SGLT2 inhibitor	362.483	-609.547-1334.512	0.458	728.824	-51.954-1509.601	0.067
Use of anti-hypertensive agent	1376.373	493.991	0.007*	782.397	-5.228-1570.023	0.051
Use of statin	890.693	41.994-1739.393	0.040*	308.355	-363.060-979.770	0.361

BMI, body mass index; SD, standard deviation; HbA1c, glycated hemoglobin A1c; BUN, blood urea nitrogen; Cr, creatinine; eGFR, estimated glomerular filtration rate; ACR, albumin to creatinine ratio; SGLT-2, sodium glucose co-transporters. *Denotes P value < 0.05.

### 3.4 Risk factors for CKD progression in study population with T2D

Among 58 subjects with diabetes, one individual initiated regular hemodialysis during the follow-up period, and three were already receiving regular hemodialysis before enrollment. After excluding these cases, 54 subjects with diabetes continued and underwent renal function follow-up, with an average follow-up duration of 16.05 ± 4.65 months. Within this group, seven individuals (12.9%) developed CKD progression, including three in CKD stage 3, two in CKD stage 4, and two in CKD stage 5). Notably, in subjects with diabetes and CKD stages 1 and 2, no renal function progression during the follow-up duration was observed.

**Table 3** displays the demographic characteristics of subjects with diabetes, both with and without CKD progression. In short, patients with CKD progression had higher levels of blood osmolarity (305.43 ± 4.35 vs. 300.93 ± 6.38 mosm/KgH2O, *P* < 0.001), creatinine (2.47 ± 1.10 vs. 1.82 ± 1.53 mg/mL, p<0.001), and lower hemoglobin (11.43 ± 1.52 vs. 13.05 ± 2.34 g/dL, *P <*0.001); additionally, the progression group had higher adropin (7520.15 ± 843.21 vs. 6151.16 ± 1661.61 pg/mL, *P* =0.003) and lower eGFR (29.37 ± 14.74 vs. 59.85 ± 41.68 mL/min/1.73m^2^, *P* < 0.001). No significant difference was found in the use of oral antidiabetic drugs or anti-hypertensive agents between the two groups.

**Table 3:** Demographic characteristics of subjects with diabetes with and without chronic kidney disease progression (Decrease eGFR >= 30%).

	CKD progression (n=7)	Non- CKD progression (n=47)	*P* value
Age (years)	68.86 ± 7.06	62.13 ± 13.41	0.273
Gender (Male), n (%)	5 (71.4%)	30 (63.8%)	0.526
BMI (kg/m2)	24.47 ± 3.70	26.50 ± 4.79	0.287
DM duration (year)	14.14 ± 4.53	11.53 ± 7.81	0.188
Osmolality (mosm/KgH2O)	305.43 ± 4.35	300.93 ± 6.38	0.039*
HbA1c (%)	7.71 ± 1.46	7.59 ± 1.69	0.495
BUN (mg/mL)	36.46 ± 17.03	27.83 ± 16.01	0.098
Cr (mg/dL)	2.47 ± 1.10	1.82 ± 1.53	0.047*
eGFR (mL/min/1.73m^2^ )	29.37 ± 14.74	59.85 ± 41.68	0.027*
Hemoglobin (g/dL)	11.43 ± 1.52	13.05 ± 2.34	0.049*
Hematocrit (%)	35.00 ± 4.11	39.83 ± 6.55	0.044*
Na (mEq/L)	140.43 ± 2.44	140.57 ± 2.61	0.969
K (mEq/L)	4.56 ± 0.49	4.61 ± 0.54	0.948
Adropin (pg/ml)	7520.15 ± 843.21	6151.16 ± 1661.61	0.023*
Urine ACR (mg/gm)	1988.54 ± 2003.85	743.65 ± 1215.79	0.098
Use of OAD			
Metformin, n (%)	3 (42.9%)	30 (63.8%)	0.256
Sulfonylurea, n (%)	6 (85.7%)	28 (59.6%)	0.182
TZD, n (%)	3 (42.9%)	11 (23.4%)	0.253
DPP4 inhibitor, n (%)	3 (42.9%)	19 (40.4%)	0.606
GLP-1R agonist, n (%)	2 (28.6%)	10 (21.3%)	0.492
SGLT2 inhibitor, n (%)	2 (28.6%)	22 (46.8%)	0.314
Insulin injection, n (%)	3 (42.9%)	12 (25.5%)	0.295
Use of anti-hypertensive agent, n (%)	7 (100.0%)	35 (74.5%)	0.152
Use of statin, n (%)	5 (71.4%)	30 (63.8%)	0.562

Continuous variables presented as mean ± standard deviation or n (%).

Mann-Whitney U test was used for continuous variables and Chi-Squared test or Fisher exact test was used for categorical variables.

*Denotes P value < 0.05.

DM, diabetes mellitus; CKD, chronic kidney disease; BMI, body mass index; HbA1c, glycated hemoglobin A1c; BUN, blood urea nitrogen; Cr, creatinine; eGFR, estimated glomerular filtration rate; ACR, albumin to creatinine ratio; OAD, oral antidiabetic drug; TZD, Thiazolidinedione; DPP4, dipeptidyl-peptidase-4; GLP-1R, glucagon-like peptide-1 receptor; SGLT-2, sodium glucose co-transporters 2.

### 3.5 ROC analyses of adropin levels and CKD progression

**Figure 3:**
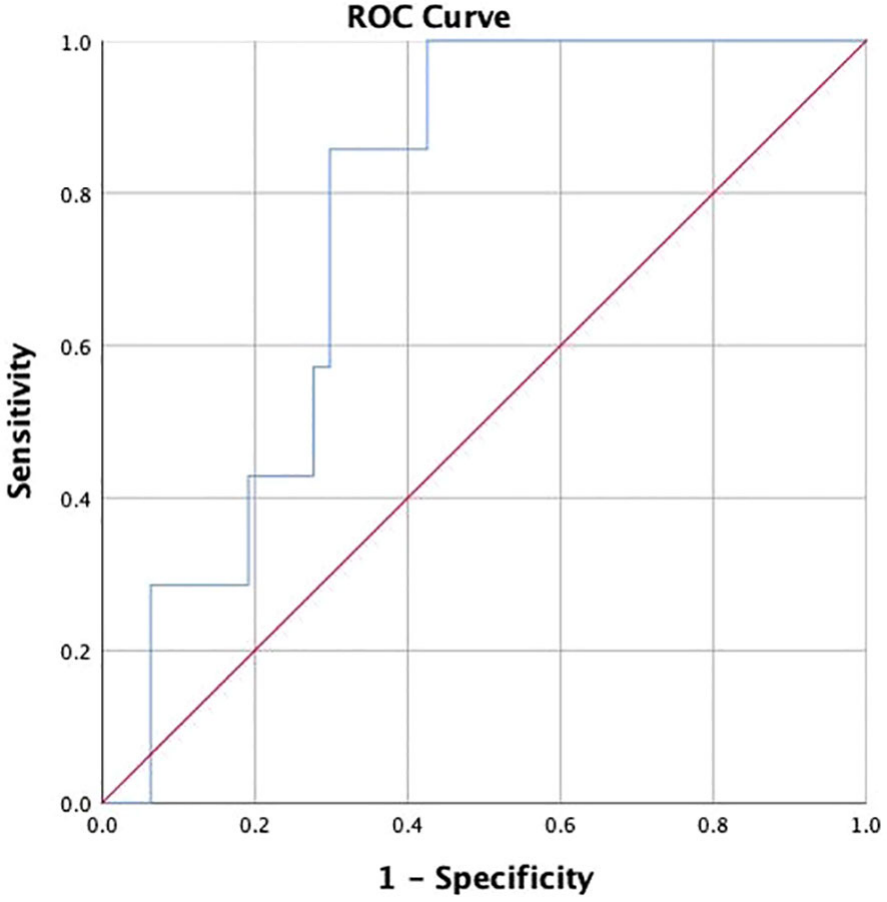
The receiver operating characteristic curve of Adropin for chronic kidney disease progression.

ROC curve analysis was performed to evaluate whether adropin could perform as a novel biomarker for distinguishing CKD progression in DKD patients. The results are shown in **Figure 3**, with an area under the curve (AUC) of 0.769 (95% confidence interval (CI) 0.635-0.903), indicating a significant predictive ability (*P*=0.0001). Notably, a cut-off value of 6872.24 pg/ml was identified (sensitivity 85.7%, specificity 70.2%, positive predictive value 74.20%, negative predictive value 80.08%), representing the optimal threshold on ROC curve for predicting renal endpoint.

### 3.6 The association of serum adropin concentrations with CKD progression

In order to evaluate the clinical value of serum adropin level in predicting CKD progression, logistic regression analysis was performed (**Table 4**), with multivariate analysis including variables such as age, gender, BMI, the cut-off point level of adropin, eGFR, serum creatinine, UACR, macroalbuminuria at baseline, the use of antihypertensive agents, SGLT2 inhibitors, and statins. Results are summarized in **Table 4**.

**Table 4:** Association between fasting high adropin concentrations (> 6872.24 pg/ml) and development of chronic kidney disease progression.

	Multivariate		
	Adjusted ORs	95% CI	*P* Value
a) Adjustment for age, gender, and BMI	27.188	1.415-522.527	0.029*
b) Adjustment as a) plus serum creatinine at baseline	23.003	1.121-471.776	0.042*
c) Adjustment as a) plus eGFR at baseline	20.754	0.704-612.226	0.079
d) Adjustment as a) plus macroalbuminuria at baseline	21.968	1.085-444.659	0.044*
e) Adjustment as a) plus urine ACR at baseline	11.836	0.536-261.232	0.118
f) Adjustment as a) plus use of anti-hypertensive treatment	17.968	0.990-326.052	0.051
g) Adjustment as a) plus use of SGL2 inhibitor	23.496	1.208-456.928	0.037*
h) Adjustment as a) plus use of statin	40.208	1.389-1164.178	0.031*

*Denotes P value < 0.05.

DM, diabetes mellitus; CKD, chronic kidney disease; OR, odds ratio; CI, confidence interval; BMI, body mass index; Cr, creatinine; eGFR, estimated glomerular filtration rate; ACR, albumin to creatinine ratio; SGLT-2, sodium glucose co-transport.

Our findings revealed an association of serum adropin level > 6872.24 pg/ml and CKD progression, even after adjusting for confounding factors such as age, gender and BMI (adjusted odds ratio (aOR) 27.188, 95% CI 1.415-522.527, *P* =0.029). This association remained significant even after adjusting for serum creatinine at baseline (aOR 23.003, 95% CI 1.121-471.776, *P* =0.042), macroalbuminuria at baseline (aOR 21.968, 95% CI 1.085-444.659, *P* =0.044), use of SGLT2 inhibitors (aOR 23.496, 95% CI 1.208-456.928, *P* =0.037), and use of statins (aOR 40.208, 95% CI 1.389-1164.178, *P* =0.031), but the association became borderline significant after adjusting for the use of antihypertensive agents (aOR 17.968, 95% CI 0.990-326.052, *P* =0.051). Additionally, no significant relationship was observed between elevated adropin concentrations and CKD progression when adjusting for eGFR and UACR at baseline.

## 4 Discussion

Although initially classified as a “promoting fat burning” peptide, adropin was later confirmed to enhance glucose oxidation and utility to reduce insulin resistance. Through knockout of the *Enho* gene, Kumar et al. demonstrated that adropin-deficiency was associated with increased risk of developing insulin resistance and diseases of metabolic syndrome (16); correspondingly, clinical observation studies corroborated this study and found that reduced circulating adropin was associated with insulin resistance, gestation and type 2 diabetes (9, 17, 18). Nevertheless, not all reports fit into this rule in the real world, where high-circulating adropin level was reported especially in patients with long-standing diabetes (11, 12, 19).

Herein, we also observed elevated adropin levels in subjects with advanced diabetes compared to those without diabetes in our study. Although lowered circulating adropin levels favor the development of diabetes from the standpoint of glucose homeostasis, it has been acknowledged that chronic oxidative stress and/or inflammation may stimulate adropin overexpression in advanced and/or poorly controlled subjects with diabetes, as has been found in rheumatic arthritis and systemic sclerosis (20–22). In correspondence, lines of evidence have documented increased serum (23) and tissue (pancreas, liver, kidney, heart, brain and cerebellar) adropin immunoreactivities (13) in diabetic rats induced by STZ. Therefore, we hypothesize a U-shaped risk association of adropin during the natural course of diabetes.

The published data suggesting oxidative stress may affect adropin expression through as yet unclarified meticulous networks. However, as mentioned above, we propose that the elevated adropin levels observed in our study may reflect a compensatory response to chronic oxidative stress and inflammation process in advanced CKD. This hypothesis is supported by evidence from chronic heart failure patients, where elevated adropin correlates with inflammation markers like IL-6 (24). Adropin may act to counterbalance the inflammatory status, which helps explain its paradoxical elevation in advanced disease stages.

Another possible interruption for serum adropin level in this study is medication, and it is worth noting that a significant proportion of our subjects with diabetes (79.3%) were taking anti-hypertensive agents, while a considerable number (62.1%) were on statin treatment, and 25.9% were using SGLT2 inhibitors. Previous research suggested that patients treated with anti-hypertensive medications like amlodipine and valsartan may have increased serum levels of adropin (25); additionally, an *in vitro* study demonstrated that atorvastatin might upregulate the expression of adropin (23), while another study demonstrated that dapagliflozin, an SGLT2 inhibitor, led to an increase in adropin level by up to 26.6% (26). Accordingly, multivariate linear regression analyses was performed to assess this correlation, with results still revealing borderline significance of higher adropin levels in subjects with diabetes after adjusting potential confounding factors including the use of the aforementioned drugs. In summary, the current study found significantly elevated serum adropin levels in subjects with diabetes, especially those with CKD, compared to those without diabetes for demographic differences.

In addition to the diabetogenic effect, lower serum adropin levels in subjects with diabetes were also found to be associated with higher possibility of developing renal and cardiovascular complications (27). Although the mechanisms as to how adropin preserves normal tissue function under hyperglycemic status remains obscure, it has been found that decreased adropin secretion in diabetic rats and adropin-knockout mice is associated with overexpressed inflammatory factors such as TNF-α, IL-6, as well as reduced phosphorylation level of eNOS (28, 29), while in contrast, increased generation and secretion of adropin promoted nitrogen oxide production and up-regulation of vascular endothelial growth factor receptors, potentially preventing endothelial cell damage in diabetic nephropathy (28, 29). Taken together, these findings suggest that adropin plays a crucial role in the pathophysiological development of diabetic nephropathy.

The association of adropin and the development and progression of diabetic nephropathy has been monitored by UACR and serum creatinine in individuals with T2D in clinical practice. In a literature review, most of the studies were focused on patients with early-stage CKD (average eGFR ranging from 68.80 to 109.13 mL/min/1.73 m^2^) (17, 18, 30). To further investigate these finding in more advanced CKD, our study focused on subjects with diabetes, with 69% classified as CKD stages 4-5 (average eGFR of 52.57 mL/min/1.73 m²), and as expected, our result is in contrast to the previous reports in early CKD, and we found that patients with advanced CKD had higher adropin levels than those with early CKD. Notably, our report is also the first study to demonstrate higher serum adropin levels; that is, those exceeding 6872.24 pg/ml, as being an independent risk factor for CKD progression.

Corresponding to our finding of elevated circulating adropin in advanced CKD, animal studies have already reported increased adropin immunoreaction documented in the kidney of STZ-induced diabetic rats with the intensities increasing with diabetic severity (13, 18). It is proposed that in response to chronic insults in poorly controlled T2D with advanced CKD, over-secretion of adropin plays a vital role in preventing further deterioration of diabetic nephropathy; therefore, higher levels of adropin in this study might represent an adaptive response with a potential protective effect against the progression of CKD. Nevertheless, as it was found in an *in vivo* study showing “adropin resistance” (31, 32), it seems that a threshold of adropin-associated renal protection exists, so when the environmental condition is exacerbated, renal function deterioration commences even when serum adropin is persistently high, just as it was observed in our progression DKD patients.

It has been well documented that the progression of CKD is associated with the nuclear factor erythroid 2-related factor 2 (Nrf2) which plays critical roles in maintaining normal redox homeostasis and renal function (33). A role of Nrf2 in DKD wa*s* demonstrated by reduced translocation of Nrf2 into the nucleus and diminished protection against inflammation, fibrosis and oxidative damage in renal tissues of diabetic rat studies (34). Nevertheless, clinical treatment of stage 4 diabetic CKD patients with a synthetic Nrf2 activator, bardoxolone methyl, in the BEACON study showed the recovery of eGFR within a narrow therapeutic range, accompanied by certain unfavorable side effects (35, 36). To integrate these findings into our study, we propose an Nrf2-mediated mechanism for “adropin resistance” in our study based on the following studies. Firstly, the naturally occurring endogenous Nrf2 activator, adropin bioactive peptides stimulated and activated Nrf2 in a dose-dependent manner in an adropin-knockout mice to prevent non-alcoholic steatohepatitis (37). Secondly, biphasic Nrf2 expression in CKD patients were reported in Denmark (38) and Italian (39) studies, where increased Nrf2 expression was detected in patients with mild to moderate renal impairment, whereas significant reduced Nrf2 was observed as CKD progressed in patients with severe renal impairment. To summarize, initially overexpressed adropin may protect against CKD progression through its dose- dependent induction and activation of Nrf2 until this protective mechanism is overwhelmed in more deteriorated conditions. Subsequently, Nrf2 exhaustion led to renal function decline and rapid progression, even with their serum adropin levels exceeding 6872.24 pg/ml.

Although we did not perform kidney biopsy or urinary analysis to check for the renal expression of adropin, Nrf2 and inflammatory markers, our results echo the findings of increased renal expression of adropin in STZ-induced diabetic rats (13, 40), which also highlights the complexity of the role of adropin in diabetic nephropathy and suggests that further research is needed to better understand its mechanisms and potential therapeutic implications.

Another important finding was the elevated osmolality in the subjects with diabetes and CKD, which was also well-correlated with the circulating adropin. In addition to the elevated blood glucose and urea nitrogen in the subjects with poorly controlled diabetes, there is a possibility that an elevated circulating adropin might also act on the brain to inhibit water intake (41, 42) cannot be excluded, and the higher osmolality might in part have contributed to the rapid progression of renal function in our DKD patients (43).

**Figure 4:**
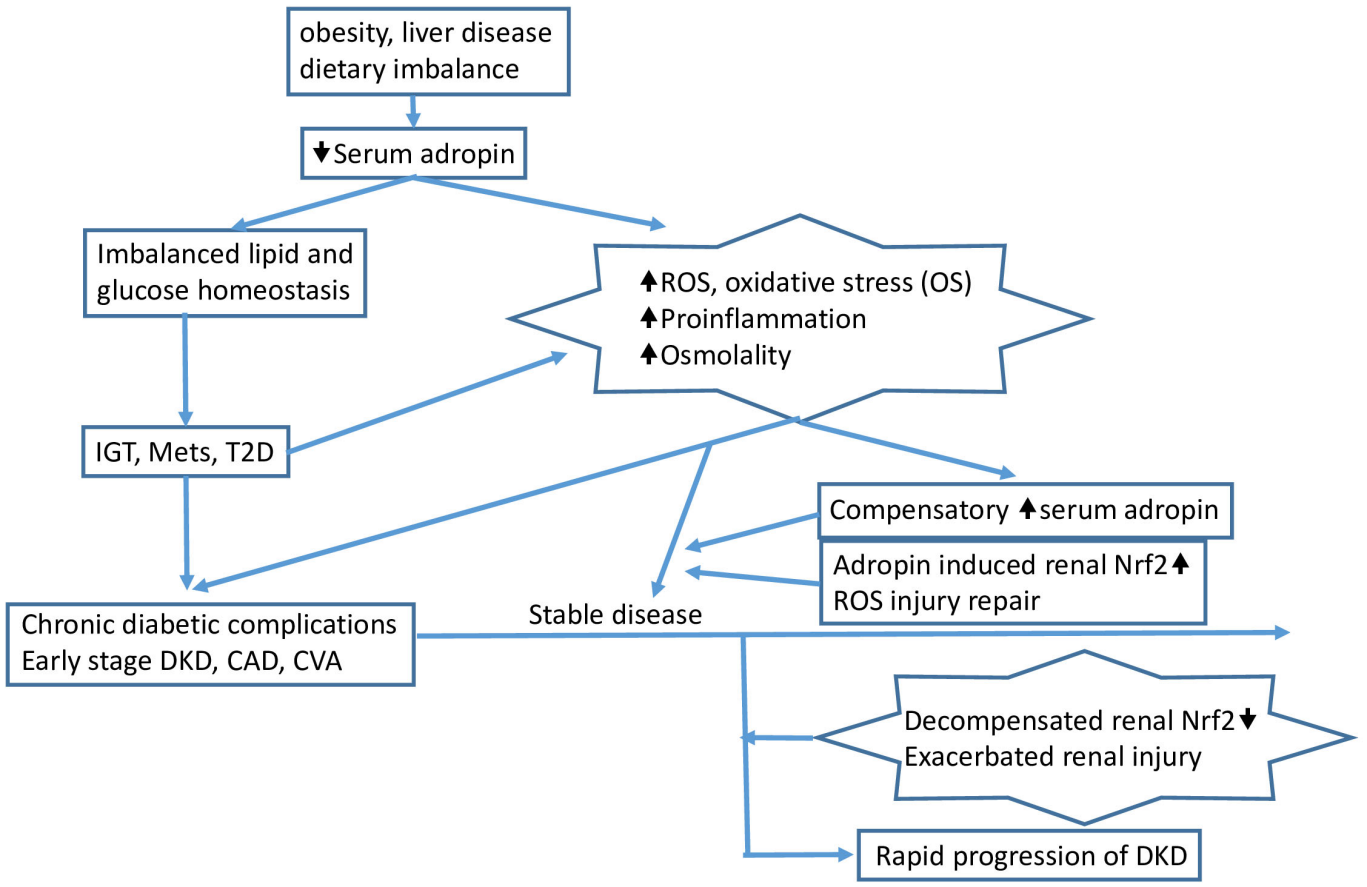
Pathophysiologic insults such as obesity, dietary imbalance, and certain liver diseases (e.g., fatty liver) reduce serum adropin and induce impaired glucose tolerance (IGT), metabolic syndrome (MetS), and/or type 2 diabetes (T2D). Accumulated increased serum glucose induces reactive oxygen species (ROS), oxidative stress (OS), proinflammation, and increased serum osmolality, leading to chronic complications in patients with advanced T2D. The proinflammatory process and/or other mechanisms induce compensatory overexpression of adropin, which then triggers renal Nrf2 overexpression, protecting the kidney from injury caused by increased ROS and proinflammation. As the environment exacerbates, Nrf2 function is compromised, and at this point, rapid progression of CKD can be observed clinically, accompanied by elevated serum adropin. The arrows indicate the sequential flow of events leading to the development of diabetic kidney disease (DKD).

These findings suggest that serum adropin levels could potentially serve as a useful biomarker for predicting rapid deterioration of renal function, especially in individuals with poorly controlled T2D and pre-existing CKD. Several reviews and the hypothesis have well summarized the evidence to date on adropin in the development of diabetes and its complications, as displayed in **Figure 4**.

This study has some limitations. Firstly, the sample size was relatively small, necessitating further studies for confirmation, while adropin concentrations were measured only under fasting conditions, which might not fully capture the fluctuations of adropin level(s) over time, particularly after different dietary intakes. Additionally, anthropologic parameters such as age, gender and body composition might also influence circulating adropin levels, while exploring the kinetics of adropin clearance from the circulation desirably measuring its concentration in various conditions could further help to clarify the possible mechanisms involved in the response of adropin to altered metabolic status. Additionally, we could not exclude the possibility that reduced nephron activity and impaired renal clearance may contribute to accumulated circulating adropin in our advanced CKD patients as a possible mechanism albeit whether adropin was excreted by urination was unclear. Finally, multicenter studies with larger sample sizes are necessary to validate our findings.

## 5 Conclusions

Despite the association between lack of circulating adropin and the development of diabetes and its renal and cardiovascular complications, this study observed significantly higher serum adropin levels in diabetes subjects with advanced nephropathy compared to those with mild diabetic nephropathy and subjects without diabetes in this study. These findings suggest that compensatory elevated adropin could be associated with proinflammation- and redox imbalance-induced renal dysfunction in patients with T2D. Adropin could potentially serve as a biomarker for early detection of renal Nrf2 insufficiency and diabetic nephropathy deterioration, especially in patients with advanced CKD. Further research is needed to better understand the exact role and mechanisms of adropin in the pathogenesis of diabetes and diabetic kidney disease. Such understanding can provide valuable insights for the development of targeted interventions and treatments in the future.

## Data Availability

The raw data supporting the conclusions of this article will be made available by the authors, without undue reservation.
